# Altered folate metabolism disrupts auditory function from neonatal vocalizations to adult perceptual precision

**DOI:** 10.1186/s11689-026-09692-2

**Published:** 2026-04-10

**Authors:** Danielle Barda, Lior Dor, Hila Sapir, Shaked Weinberger, Yuval Avni Mann, Aviv Or-Zeid, Netta Baram, Dror Lederman, Hava Golan, Jennifer Resnik

**Affiliations:** 1 Department of Physiology and Cell Biology, Ben-Gurion University of the Negev, Beer Sheva, 84105 Israel; 2 Department of Life Sciences, Ben-Gurion University of the Negev, Beer Sheva, 84105 Israel; 3 Zelman Center for Brain Science Research, Ben-Gurion University of the Negev, Beer Sheva, 84105 Israel; 4 Faculty of Engineering, Holon Institute of Technology, Holon, 5810201 Israel

## Abstract

Methylenetetrahydrofolate‐reductase (MTHFR) is a critical enzyme in folate-dependent one-carbon metabolism. While MTHFR polymorphisms have been linked to sensorineural hearing loss and neurodevelopmental disorders in humans, the translation of early-life metabolic stress into long-term sensory processing and perceptual consequences has remained unclear. Here, we combined neonatal ultrasonic vocalization analysis, adult auditory-brainstem recordings, two-photon calcium imaging of the auditory cortex, and high-resolution psychophysics to map the effects of partial Mthfr deficiency across different developmental stages. MTHFR-deficient pups produced calls with lower onset frequencies and altered temporal structure. In adulthood, MTHFR-deficient mice exhibited elevated auditory brainstem response thresholds and reduced wave I amplitudes, indicating reduced cochlear output. Despite robust cortical tone responses, two-photon imaging revealed significantly broadened frequency–response areas, and a trained cortical activity decoder showed selectively reduced separability for closely spaced tones. Guided by these neural findings, behavioral testing revealed intact discrimination for widely separated tones but impaired performance near the perceptual boundary, indicating reduced fine spectral acuity. Together, these findings outline a developmental trajectory in which early metabolic stress impairs peripheral encoding, central gain compensates for the loss of sensitivity, and perceptual precision is ultimately compromised, highlighting one-carbon dysregulation as a modifiable contributor to auditory dysfunction.

**Supplementary Information:**

The online version contains supplementary material available at 10.1186/s11689-026-09692-2.

## Introduction

Auditory function is assembled through a tightly timed developmental program in which peripheral sensitivity, central gain, and perceptual resolution are calibrated in early life and then refined with experience. Because this calibration is sensitive to metabolic state, even mild developmental constraints can bias the system toward compensatory solutions that preserve audibility but compromise fidelity under high perceptual demand.

Folate-dependent one-carbon metabolism is fundamental for nucleotide synthesis, methyl-group supply, and redox balance. A bottleneck enzyme in this pathway, methylenetetrahydrofolate-reductase (MTHFR), remethylates homocysteine to methionine; hypomorphic MTHFR variants or dietary folate deficiency therefore elevate homocysteine and oxidative stress—two insults that selectively damage the metabolically demanding cochlea. In humans, the common MTHFR C677T allele is over-represented in sudden sensorineural hearing-loss cohorts [24, 36] and several human studies link higher homocysteine levels to sudden or progressive sensorineural hearing loss [12, 22]. Parallel work in mice shows that dietary folate deprivation or genetic reduction of one-carbon flux accelerates ribbon-synapse loss, elevates auditory-brainstem-response (ABR) thresholds, and shortens the time-course of hearing senescence [20]. Together, converging clinical and pre-clinical data position impaired MTHFR activity as a potentially modifiable molecular driver of cochlear pathology.

A key feature of many developmental hearing disorders is that functionally relevant deficits can emerge very early, often before reliable age-appropriate clinical hearing tests can be performed, limiting our ability to capture impairment at the earliest stages. Classic examples include GJB2/Connexin-26 [18] and PCDH15 [1] mutations, which produce congenital or early-postnatal deafness in both children and mouse models. Evidence for MTHFR mirrors this pattern: newborn pups carrying the Mthfr-knock-out allele emit isolation calls with lower peak frequencies and altered syntax—acoustic fingerprints consistent with altered early-life vocal output [35]. Although pup (ultrasonic vocalizations) USVs are influenced by factors such as arousal and thermoregulation, systematic shifts in call spectral structure provide an early, behaviorally accessible window into developmental sensory constraints when electrophysiological testing is limited. Case–control studies further report that children with inherited disorders of homocysteine metabolism (including severe MTHFR deficiency) frequently present with delayed auditory-brainstem maturation and elevated click thresholds, underscoring the developmental reach of one-carbon stress [11].

Chronic reduction of peripheral input seldom remains a silent lesion: the auditory brainstem and cortex frequently raise their neural gain to stabilize activity. In rodents, noise-induced synaptopathy or hair-cell loss drives hyperactivity along the pathway, producing steeper loudness growth and broader tuning despite only modest threshold shifts [2, 27, 34]. Such homeostatic compensation can mask the true extent of cochlear damage while degrading fine spectral resolution. Given that MTHFR insufficiency perturbs cochlear metabolism from gestation onward [35], a similar peripheral-to-central cascade is plausible; consistent with this framework, we found recently that adult Mthfr ± (MTHFR-deficient) mice display cortical hyper-responsivity [32], suggesting compensatory gain changes that may ultimately become maladaptive for fine perception.

These observations suggest that impaired one-carbon metabolism may not simply reduce auditory sensitivity but rather bias the developmental calibration of the auditory system, potentially engaging compensatory mechanisms that preserve audibility while compromising resolution. To test whether partial MTHFR deficiency biases the developmental calibration of the auditory system, we examined auditory function across key maturational stages and various levels of analysis. Specifically, we recorded P6 isolation-induced USVs to capture early sensory constraints and maternal-reunion–driven tuning, measured adult ABRs to assess long-lasting changes in cochlear/nerve output, we used two-photon calcium imaging of auditory cortex to quantify frequency–response area features and assess how cortical tuning supports neural separability of nearby versus distant tones, and tested adult frequency discrimination across frequencies with increasing similarity to determine whether any impairment is most evident under fine spectral acuity demands. This staged approach enabled us to investigate whether a developmental cascade initiated by early metabolic stress ultimately influences adult auditory perception.

## Results

To determine whether impaired one-carbon metabolism alters auditory function during early postnatal maturation, we recorded isolation-induced USVs from Mthfr +/– pups (MTHFR-deficient pups) at post-natal day 6 (P6) and age-matched WT controls. Because pups vocalize before the onset of robust startle or ABR reflexes, USV analysis offers a non-invasive window into early cochlear integrity and the developmental impact of metabolic stressors such as MTHFR deficiency. Using the maternal enhancement of mouse pup isolation calls protocol, two brief five-minute recording sessions were obtained from each animal—before and after a 20-min reunion with the dam—yielding 6,184 calls from male pups and 6,366 from female pups (6,216 MTHFR-deficient; 6,434 WT).

**Fig. 1:**
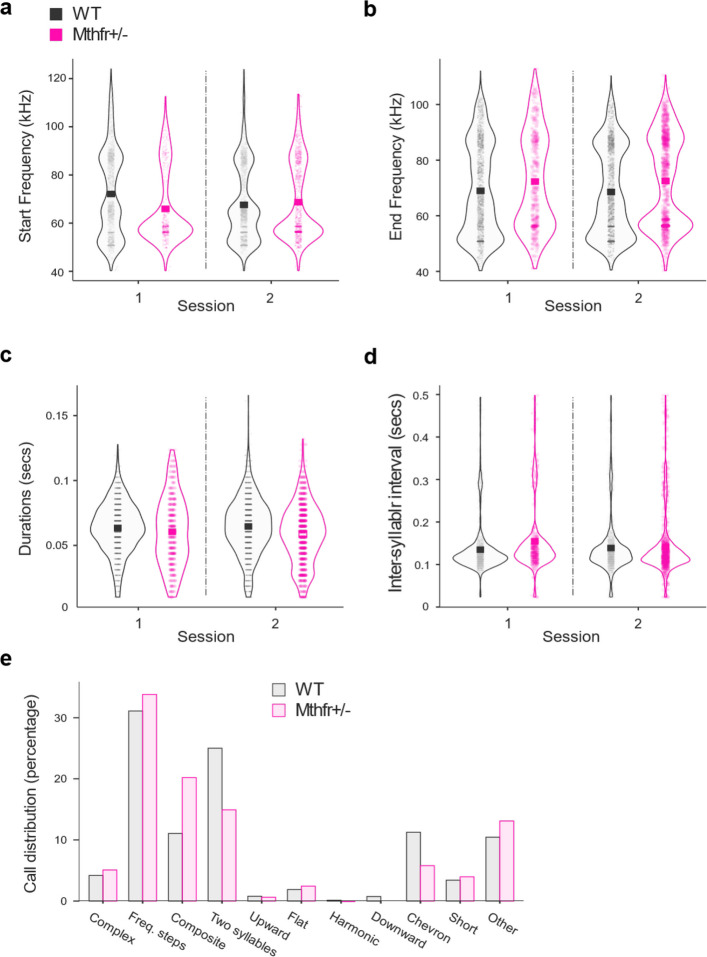
MTHFR-deficiency altered the spectral and temporal features of isolation calls in male mouse pups. **a** Distribution of start frequencies for isolation calls emitted by MTHFR-deficient (pink) and wild-type (WT, black) pups. MTHFR-deficient pups initiated calls at significantly lower frequencies than WT pups (2-way ANOVA, genotype effect: F = 24.24, *p* < 0.00001). **b** Distribution of end frequencies. MTHFR-deficient pups exhibited slightly higher end frequencies compared to WT (2-way ANOVA, genotype effect: F = 75.83, *p* < 0.00001). **c** Call duration was consistently shorter in MTHFR-deficient pups across sessions (2-way ANOVA, genotype effect: F = 48.36, *p* < 0.00001). **d** Inter-syllable intervals (ISI) were significantly longer in MTHFR-deficient pups (2-way ANOVA, genotype effect: F = 36.42, *p* < 0.00001). **e** Call-type distribution differed between genotypes. MTHFR-deficient pups produced more composite calls and fewer two-syllable and chevron calls compared to WT pups (χ.^2^ test, *p* < 0.001). For panels (**a**–**d**), mean values are indicated by black (WT) or pink (MTHFR-deficient) squares. Data is based on 4,471 calls from 8 WT pups and 1,813 calls from 3 MTHFR-deficient pups. Complete results of the post-hoc analysis and effect sizes are presented in Supplemental Table 1

Across sessions, males call rate did not differ significantly between genotypes; WT pups emitted 258 ± 68 and 300.8 ± 59 calls per pup in sessions 1 and 2, respectively, whereas MTHFR-deficient pups emitted 176 ± 39 and 302 ± 73 calls per pup in sessions 1 and 2, respectively, which did not significantly differ between genotypes or sessions. While there was a similar number of calls per pup, we found significant differences in their spectral and temporal features. MTHFR-deficient pups launched their calls at lower start frequencies than WT pups (Fig. 1a, 2-way ANOVA genotype F = 24.24, *p* < 0.00001), whereas their end frequencies were higher (Fig. 1b, 2-way ANOVA genotype F = 75.83, *p* < 0.00001); a significant genotype and session interaction revealed that start frequency drifted upward in MTHFR-deficient pups but downward in WT pups between the first and second isolation periods (2-way ANOVA genotype x session interaction F = 50.3, *p* < 0.00001. Complete statistics are presented in the Supplemental Table 1).

**Fig. 2:**
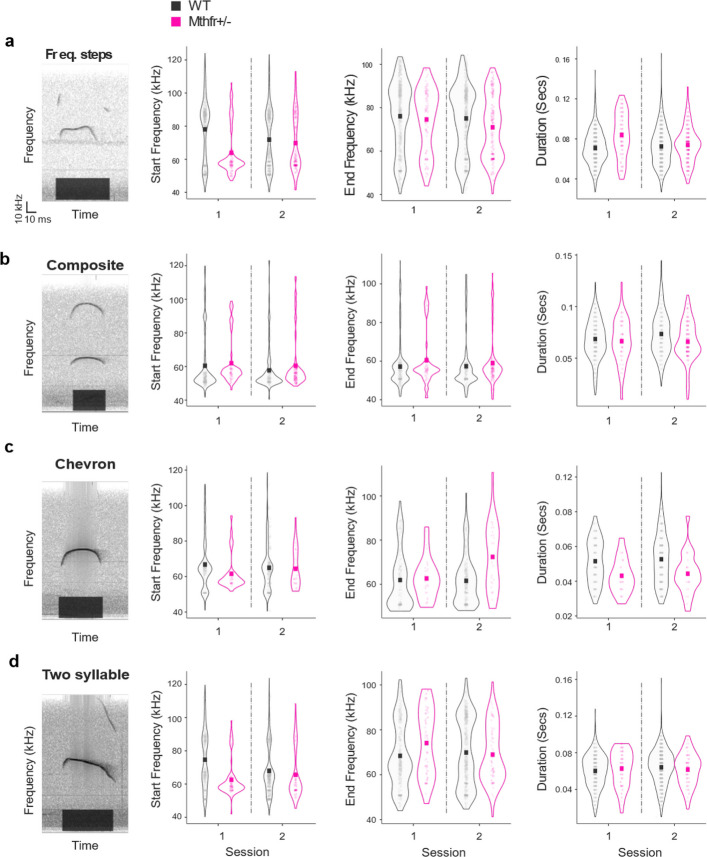
MTHFR deficiency differentially alters the spectral and temporal features of common call types. Representative sonograms of four common calls are shown on the left. The black bar at the bottom of the calls marks the detection of the call from start to end. Start frequency, end frequency, and duration are shown for (**a**) frequency-step (an abrupt shift between two relatively stable frequency bands within a single call), (**b**) composite (two harmonically independent components, emitted simultaneously), (**c**) chevron (characteristic rise and then fall in frequency), and (**d**) two-syllable (a main call (flat or downward) with an additional punctuated component towards the end). Calls produced by MTHFR-deficient (pink) and wild-type (WT, black) pups. Mean values are indicated by pink (MTHFR-deficient) or black (WT) squares. MTHFR-deficient pups initiated frequency-step and two-syllable calls at lower frequencies (**a**, **d**; left panels) and ended composite and chevron calls at higher frequencies (**b**, **c**; middle panels) compared to WT pups. Call durations were longer in MTHFR-deficient pups for frequency-step calls (**a**; right panel) but shorter for composite and chevron calls (**b**, **c**; right panels), with several effects influenced by recording session. Call counts: frequency-step (*N* = 1786), composite (*N* = 693), chevron (*N* = 560), two-syllable (*N* = 1265). Scale bar: 10 kHz and 10 ms. Extended post-hoc analysis and effect sizes are presented in Supplemental Table 2

The call’s temporal variables were also significantly different. Call duration in male pups was consistently shorter in MTHFR-deficient pups regardless of recording session (Fig. 1c, 2-way ANOVA genotype F = 48.356, *p* < 0.00001), and inter-syllable intervals (ISI) were longer (Fig. 1d, 2-way ANOVA F = 36.42, *p* < 0.00001), again with an interaction indicating opposite within-session shifts in the two genotypes (2-way ANOVA session, F = 5.700, *p* = 0.017, genotype x session, F = 19.38, *p* < 0.00001). So that in the MTHFR-deficient pups, ISI was shorter in session 2 compared to session 1, and in the WT pups, ISI was longer in session 2 compared to session 1. This mirrored the divergent start-frequency shift described above, emphasizing that MTHFR-deficient and WT pups exhibit opposite experience-dependent tuning following the brief maternal reunion.

Finally, the distribution of call types differed: MTHFR-deficient mice produced a distinct vocal repertoire compared with WT pups. Composite and frequency-step calls were over-represented, whereas two-syllable and chevron calls were under-represented (Fig. 1e, χ^2^, *p* < 0.001). Since frequency-step, composite, two-syllable, and chevron calls accounted for most of the dataset and displayed the most pronounced genotype-linked shifts, we next examined whether their detailed acoustic structure was also affected.

In frequency-step calls, MTHFR-deficient pups began and ended the call at lower frequencies than WT pups (Fig. 2a left and middle, 2-way ANOVA start: F = 49.6, *p* < 0.00001; end: F = 8.04, *p* = 0.0046). The reduction in start frequency interacted with recording session (2-way ANOVA session x genotype F = 27.3, *p* < 0.00001), whereas the change in end frequency was accompanied by a modest main effect of session common to both genotypes (2-way ANOVA F = 5.39, *p* = 0.020). Composite calls opened at similar frequencies in the two groups but ended at a higher frequency in MTHFR-deficient pups (Fig. 2b, middle, 2-way ANOVA F = 4.61, *p* = 0.032) with no session influence. Chevron calls showed comparable start frequencies, yet their end frequency was elevated in MTHFR-deficient pups (2-way ANOVA F = 11.86, *p* = 0.00062, with a Cohen D = 0.487) and was further modulated by session (Fig. 2c, middle, 2-way ANOVA F = 7.91, *p* = 0.005) with a significant genotype-by-session interaction (2-way ANOVA session x genotype F = 9.24, *p* = 0.002). The strong effect of this interaction is demonstrated by Choen’s D above 0.8 as presented in Supplemental Table 2. Two-syllable calls began at a lower frequency in MTHFR-deficient pups (Fig. 2a left, 2-way ANOVA F = 29.07, *p* < 0.00001, Cohen’s D = 0.475, with a stronger effect size during the first session, Choen’s D = 0.795) but showed only a trend toward a higher end frequency (2-way ANOVA F = 3.34, *p* = 0.068); both start and end frequencies interacted with session (2-way ANOVA start: F = 13.17, *p* = 0.0003; end: F = 6.46, *p* = 0.011).

Temporal parameters were likewise call-specific. Frequency-step calls were longer in MTHFR-deficient pups (Fig. 2a right, 2-way ANOVA F = 37.8, *p* < 0.00001) and lengthened between sessions (2-way ANOVA F = 12.9, *p* = 0.00035), with the two factors interacting (F = 24.2, *p* < 0.00001). Composite and chevron calls were shorter in MTHFR-deficient pups (Fig. 2b and c right, composite: 2-way ANOVA: F = 7.28, *p* = 0.0072; chevron: 2-way ANOVA: F = 19.24, *p* < 0.00001, Cohen’s D = 0.62) and were unaffected by session, whereas two-syllable call duration remained unchanged across genotypes and sessions (Fig. 2d right).

When we examined the female pup calls (1963 calls emitted by 4 WT and 4403 by 6 MTHFR-deficient female pups), we found a similar pattern in spectral but not temporal aspects of the calls. MTHFR-deficient females initiated their calls at lower start frequencies than WT pups (Sup Fig. 1a, 2-way ANOVA genotype F = 8.657, *p* = 0.00327), and this start frequency rose between the first and second isolation periods, whereas it fell in WT pups (2-way ANOVA genotype x session interaction F = 33.308, *p* < 0.00001). Their end frequencies were slightly lower than WT in the first session but shifted upward in the second (Sup Fig. 1b, 2-way ANOVA genotype x session interaction F = 15.670, *p* = 0.00008).

The call’s temporal variables were also significantly different between WT and MTHFR-deficient female pups, but in an opposing direction, relative to the male pups. Call duration in female pups was consistently longer in MTHFR-deficient pups, with an effect of the recording session and slight interaction between genotype and (Sup Fig. 1c, 2-way ANOVA genotype F = 24.470, *p* < 0.00001, session F = 29.37, *p* < 0.00001 and genotype x session F = 4.180, *p* = 0.04102), and inter-syllable intervals (ISI) were shorter in the MTHFR-deficient compared to the WT pups (Sup Fig. 1 d, 2-way ANOVA F = 17.223, *p* = 0.00003).

Together, these results show that partial loss of MTHFR function alters pup communication at an early developmental stage: MTHFR-deficient male pups emit calls that begin at lower frequencies, are shorter, have longer inter-syllable intervals, and favor composite over two-syllable and chevron call types. Female pups also emit calls that begin at lower frequencies but are longer and have a shorter inter-syllable interval. In both sexes, the opposing session-dependent shifts—MTHFR-deficient pups raising their start frequency and shortening inter-syllable gaps after the brief dam reunion, whereas WT pups show the reverse—indicate that the experience-dependent acoustic plasticity of isolation calls is altered: MTHFR deficiency skews how maternal contact fine-tunes the spectral and temporal structure of the vocalizations.

The acoustic structure of neonatal USVs can provide an early behavioral signature of atypical auditory system development. The spectral and temporal changes we observed in MTHFR-deficient pups prompted us to investigate whether early-life alterations in vocal output are associated with a persistent reduction in peripheral auditory function into adulthood.

**Fig. 3:**
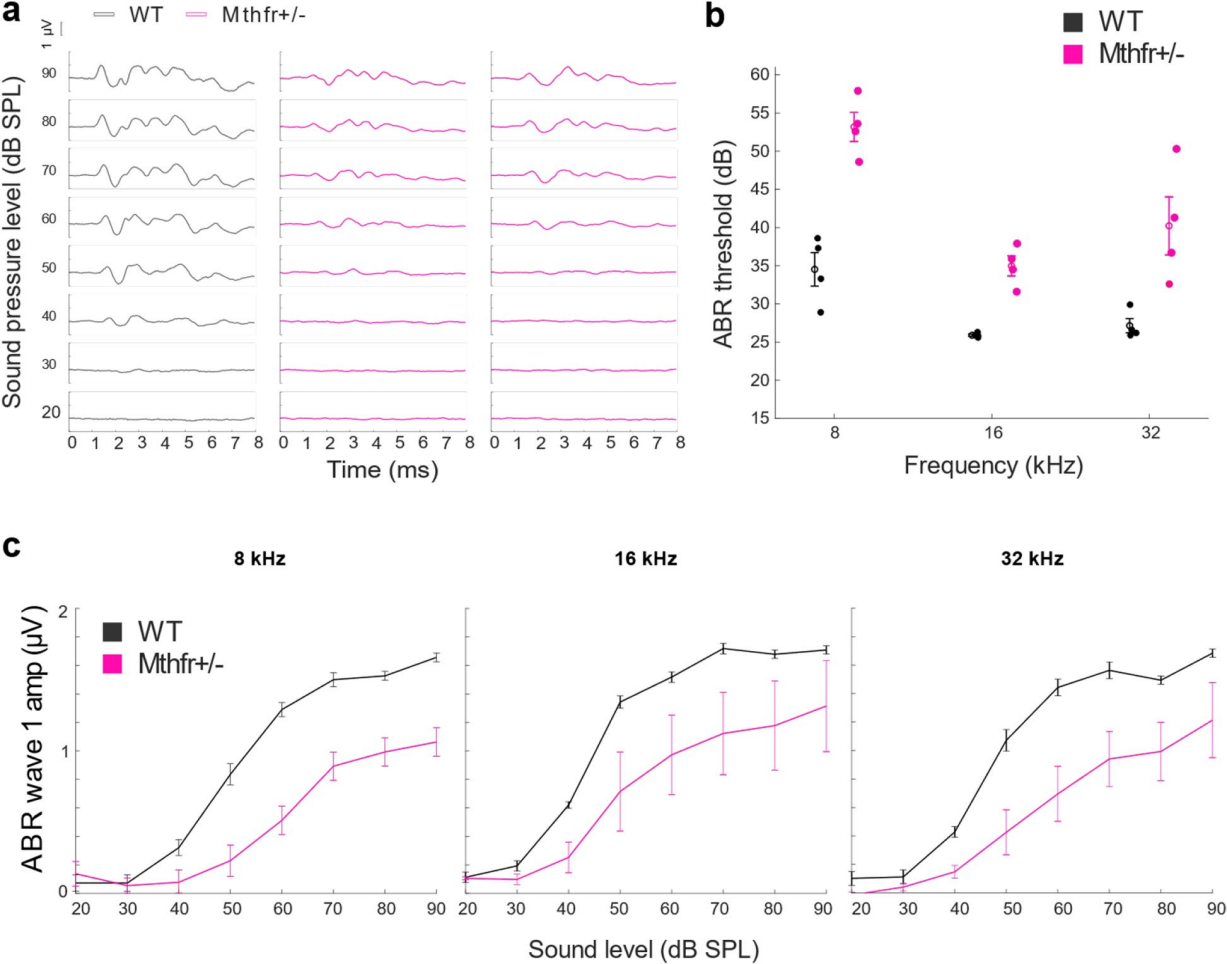
MTHFR deficiency reduces auditory sensitivity in adult mice. **a** Representative auditory brainstem response (ABR) waveforms from one WT and two MTHFR-deficient adult mice. **b** ABR thresholds were significantly elevated in MTHFR-deficient mice across all tested frequencies (8, 16, and 32 kHz; 2-way ANOVA, genotype effect: F = 65, *p* = 2.1 × 10⁻⁷). Data are presented as mean (open circles) ± SEM. **c** ABR wave 1 amplitudes were significantly reduced in MTHFR-deficient mice at each frequency (8 kHz: F = 120, *p* = 1.1 × 10⁻^14^; 16 kHz: F = 19.18, *p* = 0.0001; 32 kHz: F = 51, *p* = 4.4 × 10⁻⁹). Data are presented as mean ± SEM

To test whether MTHFR deficiency impairs auditory function, we measured auditory brainstem responses (ABRs), which assess the neural activity of the auditory nerve and brainstem pathways in response to sound, in MTHFR-deficient adult mice (Fig. 3a, *n* = 4) and age-matched WT controls (*n* = 4). Because we observed that neonatal USV features (and their genotype-linked shifts) differ by sex, we conducted subsequent experiments in adult males to follow a single, consistently affected cohort. MTHFR-deficient mice exhibited significantly higher ABR thresholds compared to WT controls in all frequencies tested (Fig. 3b, 2-way ANOVA genotype F = 65 *p* = 2.1 × 10^–07^, interaction F = 2.7 *p* = 0.09), aligning with elevated ABR thresholds as a result of dietary folate deprivation [20]. Specifically, MTHFR-deficient mice showed an average threshold elevation of 10 dB for 16 kHz, 13 dB for 32 kHz, and 18 dB for 8 kHz. We also observed decreased wave I amplitudes across all frequencies (Fig. 3c, 2-way ANOVA genotype 16 kHz F = 19.18 *p* = 0.0001, 32 kHz F = 51 *p* = 4.4 × 10^–09^, and 8 kHz F = 120 *p* = 1.1 × 10^–14^). These findings suggest a marked reduction in auditory sensitivity in MTHFR-deficient mice, usually associated with impaired auditory nerve function and reduced output from the early stages of auditory processing.

**Fig. 4:**
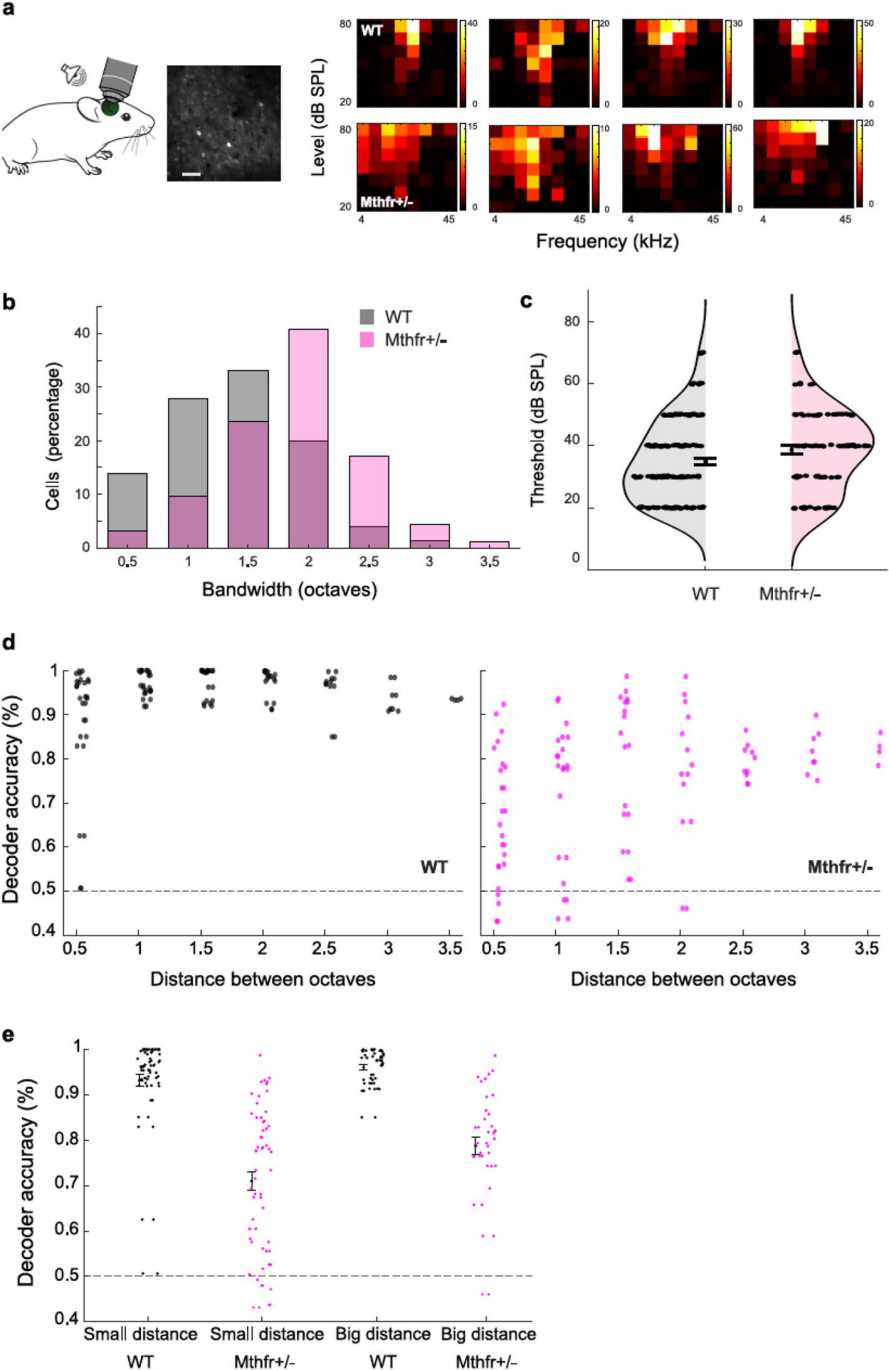
Wider tuning curves predict impaired discrimination for tones with small octave separations in MTHFR-deficient mice **a** Left: Experimental schematics and a representative imaging plane from a GCaMP-injected mouse. Mice listened to pure tones with different frequency and intensity combinations (*N* = 4 WT and 4 MTHFR-deficient mice, *n* = 586 and 550 accordingly). Frequency response areas (FRAs) were computed for each imaged cell, and FRA response quality was quantified using d′ [13]. Unless otherwise noted, subsequent receptive-field analyses include only cells with well-defined FRAs (d′ > 1.5, *n* = 170 WT cells and *n* = 154 MTHFR-deficient cells). Right: example of frequency response areas for four cells from a WT mouse and four cells from an MTHFR-deficient mouse. **b** FRA bandwidth 10 dB above threshold was wider in MTHFR-deficient mice than in WT mice (KS-test *p* = 3.3 × 10^⁻52^, t-test *p* = 6.03 × 10^⁻60^). **c** Best frequency thresholds were slightly higher in MTHFR-deficient mice than in WT mice (KS-test *p* = 0.019, t-test *p* = 0.029). Data are presented as mean ± SEM. **d** Classification of frequencies based on cell activity at 70 dB SPL, divided into bins by the distance between the discriminated frequencies, per session for MTHFR-deficient and WT mice. MTHFR-deficient activity-based decoder showed a reduced accuracy (2-way ANOVA group F = 80.5 *p* = 2.4 × 10–16, octave separation F = 4.6 *p* = 1.7 × 10^–4^) for close frequencies (post hoc 0.5 octave separation *p* = 3.55 × 10^–09^), but accuracy similar to the WT activity-based decoder for bigger separation between frequencies (post hoc 3 octave separation *p* = 1). **e** Discrimination of close frequencies (1.5 octaves or less) and frequencies further apart (more than 1.5 octaves difference) per session for MTHFR-deficient- and WT-based cell activity decoder. MTHFR cells showed a lower decoding accuracy than WT cells (2-way ANOVA group F = 142.2 *p* = 2.9 × 10^–25^, Freq F = 10.2 *p* = 0.001). Interestingly, the WT-based decoder showed high accuracy starting at close frequencies and had very little room for improvement, while the MTHFR-based decoder started with a moderate accuracy and improved for frequencies further apart (post hoc small MTHFR-deficient vs WT *p* = 3.8 × 10^–20^, post hoc big MTHFR-deficient vs WT *p* = 1.4 × 10^–10^, WT small vs WT big *p* = 1, MTHFR-deficient small vs big *p* = 0.009, Bonferroni corrected for multiple comparisons). Data are presented as mean ± SEM

Elevated ABR thresholds and reduced wave I amplitudes alone do not necessarily predict impaired spectral coding. During development, central compensatory mechanisms may emerge to preserve stable sound processing and perception despite compromised auditory nerve function. We therefore asked whether these peripheral physiological impairments translate into degraded cortical sound processing in adulthood. Using two-photon calcium imaging, we recorded neural activity in layers 2/3 of the auditory cortex of MTHFR-deficient and WT mice during passive presentation of pure tones spanning multiple frequencies and intensities (Fig. 4, *N* = 4 WT and 4 MTHFR-deficient mice, *n* = 586 and 550 cells accordingly) and quantified frequency–response area (FRA) features in tone-responsive cells (Fig. 4a). As expected in WT mice [13], FRAs were well defined, exhibited low response thresholds, and showed normally distributed bandwidths (Fig. 4b-c). FRAs in MTHFR-deficient mice were similarly well defined, but displayed significantly broader bandwidths (Fig. 4b, KS-test *p* = 3.3 × 10^⁻52^, t-test *p* = 6.03 × 10^⁻60^) and slightly elevated thresholds (Fig. 4b, KS-test *p* = 0.019, t-test *p* = 0.029), with no significant difference in the tested cells’ best frequency (Sup Fig. 2a, t-test *p* = 0.21).

Wider FRA bandwidths could reduce frequency selectivity, increasing overlap in neural responses to nearby tones and thereby impairing fine frequency discrimination. To test whether this is the case in MTHFR-deficient mice, we trained an SVM decoder to discriminate between all possible tone combination pairs at 70 dB SPL (well above threshold) and quantified decoder performance as a function of the frequency separation between tones. In WT mice, decoding accuracy was high across the different frequency separations, consistent with robust cortical discriminability even for nearby tones. In MTHFR-deficient mice, however, decoding accuracy was reduced for closely spaced frequencies (Fig. 4d, 2-way ANOVA group F = 80.5 *p* = 2.4 × 10^–16^, frequency separation F = 4.6 *p* = 1.7 × 10^–4^, post hoc 0.5 octave separation *p* = 3.55 × 10^–09^), while performance converged with WT at larger separations (post hoc 3 octave separation *p* = 1). To further quantify this effect, we binned frequency pairs into “small distance” (≤ 1.5 octaves) and “big distance” (> 1.5 octaves). Across bins, decoding accuracy remained lower in MTHFR-deficient mice than in WT mice (Fig. 4e, 2-way ANOVA group F = 142.2 *p* = 2.9 × 10^–25^, frequency separation F = 10.2 *p* = 0.001). As expected, WT decoding was already near ceiling for small separations and showed minimal improvement with increasing distance. Decoding in MTHFR-deficient mice, however, started lower and improved as tones became more widely separated (post hoc small separation MTHFR-deficient vs WT *p* = 3.8 × 10^–20^, post hoc big separation MTHFR-deficient vs WT *p* = 1.4 × 10^–10^, WT small vs WT big separation *p* = 1, MTHFR-deficient small vs big separation *p* = 0.009, Bonferroni corrected for multiple comparisons). These results suggest that broadened cortical tuning in MTHFR-deficient mice selectively limits the discriminability of nearby frequencies while preserving coarse frequency discrimination, consistent with impaired fine spectral coding despite only modest shifts in response thresholds.

**Fig. 5:**
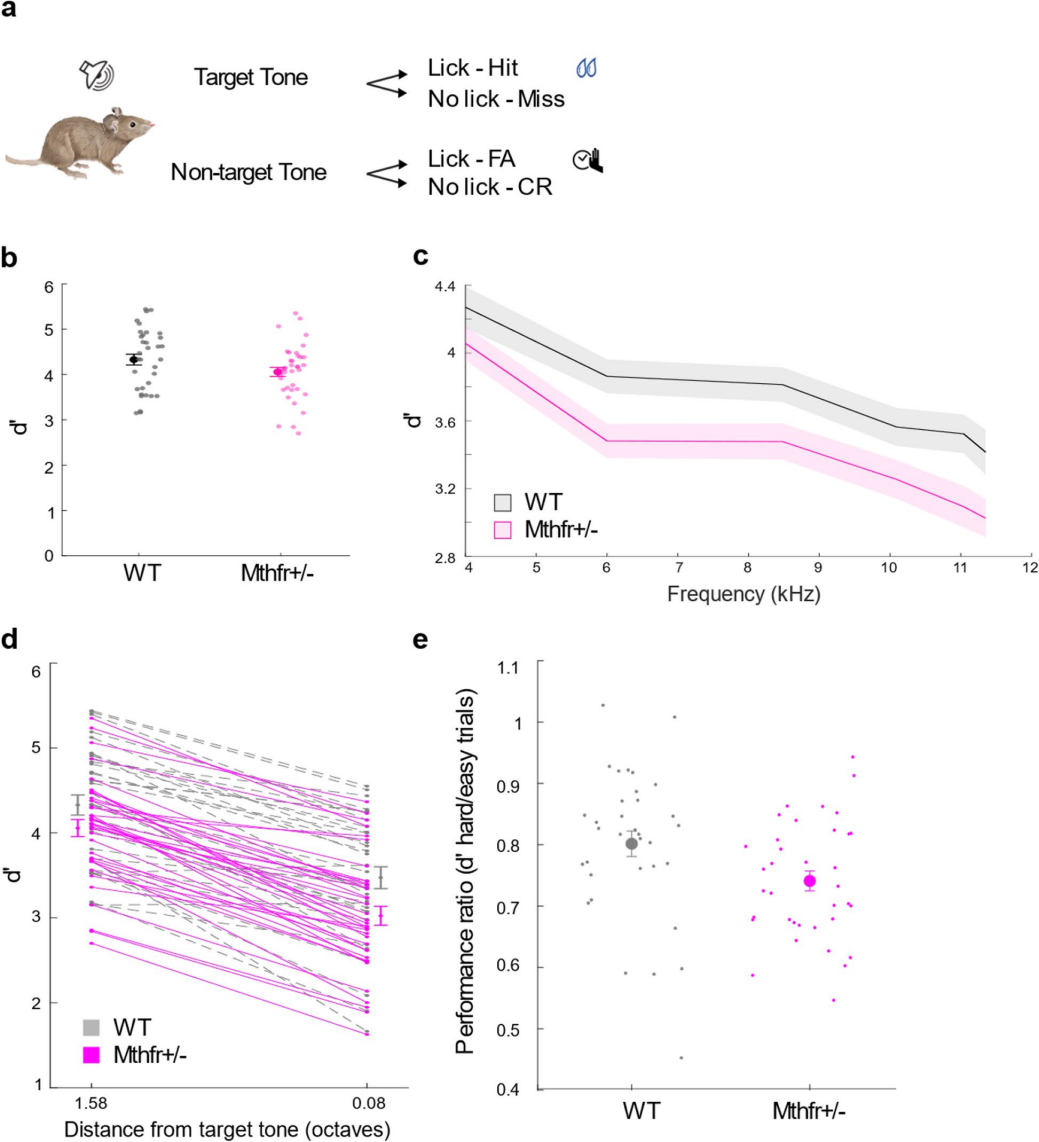
MTHFR deficiency impairs fine but not coarse auditory discrimination. **a** Schematic of the head-fixed go/no-go frequency discrimination task. Mice (4 WT and 4 MTHFR-deficient) were trained to lick in response to a 12 kHz target tone (go) to receive a water reward and to withhold licking to non-target tones (no-go). **b** Discrimination performance (d′) across trials for the trained 12 kHz (go) and 4 kHz (no-go) tones. Both groups learned the task successfully, showing robust discrimination between these widely spaced frequencies. Data are presented as mean ± SEM. **c** Performance across all non-target frequencies (4, 6, 8.48, 10.09, 11.04, 11.35 kHz). While both WT and MTHFR-deficient mice performed the task efficiently, WT mice consistently outperformed MTHFR-deficient mice (2-way ANOVA: genotype effect F = 40.9, *p* = 4.18 × 10⁻.^1^⁰; genotype × frequency interaction: F = 0.25, *p* = 0.90). Data are presented as mean ± SEM. **d** Comparison of performance on the easiest (4 kHz vs 12 kHz, 1.58 octaves apart) and most challenging (11.35 kHz vs 12 kHz, 0.08 octaves apart) conditions. Both groups achieved near-ceiling performance on the easy trials (d′ ≈ 4), with no significant genotype difference (post-hoc: *p* = 0.57). However, on the most difficult trials, MTHFR-deficient mice showed reduced performance (post-hoc: *p* = 0.03), indicating impaired fine spectral acuity. Data are presented as mean ± SEM. **e** Hard-to-easy performance ratio. MTHFR-deficient mice’s performance was less stable when transitioning from easy to hard trials (t-test *p* = 0.02)

We next asked whether this neural signature—good discriminability for distant frequencies but reduced separability for closely spaced tones—predicts a parallel perceptual deficit in vivo. To link cortical encoding to perception, we tested MTHFR-deficient and WT mice (4 per group) in a go/no-go auditory discrimination task in which non-target tones were progressively titrated closer to the target frequency. Mice were trained to lick a waterspout in response to a target tone (12 kHz at 65 dB SPL) and to withhold licking when presented with a non-target frequency (4 kHz; Fig. 5a). Both groups successfully learned the task, reaching robust discrimination (d′ > 3) with no significant difference between genotypes (Fig. 5b, t-test *p* = 0.08).

To probe frequency resolution, we then introduced non-target tones increasingly similar to the target frequency (6.00, 8.48, 10.09, 11.04, and 11.35 kHz), enabling estimation of fine frequency discrimination near the perceptual boundary. Although both groups performed efficiently, WT mice consistently outperformed MTHFR-deficient mice across the full frequency set (Fig. 5c, 2-way ANOVA, genotype: F = 40.9, *p* = 4.18 × 10⁻^1^⁰; genotype × frequency interaction: F = 0.25, *p* = 0.90), suggesting that WT controls retain sharper auditory tuning near the perceptual boundary, whereas MTHFR‑deficient mice show reduced resolution.

Notably, the difference in performance between the groups was more pronounced for non-targets closest to the target (6.00, 8.48, 10.09, 11.04, and 11.35 kHz with distances of 1, 0.5, 0.25, 0.12, and 0.08 octaves from the target tone). Whereas performance was more similar for the distant non-target (4 kHz), where the task was easier. This pattern suggests a selective deficit in fine-grained frequency discrimination in MTHFR-deficient mice, potentially reflecting reduced precision in cortical frequency encoding.

To isolate this effect, we compared the easiest (4 kHz vs 12 kHz) and most challenging (11.35 kHz vs 12 kHz) trial types. As expected, both groups performed near ceiling level on easy trials (Fig. 5d, d′ ≈ 4; 2-way ANOVA, genotype F = 9.87, *p* = 0.002; post-hoc 4 kHz WT vs MTHFR-deficient mice *p* = 0.57). In contrast, performance diverged on difficult trials that required fine resolution of closely spaced frequencies (post-hoc comparison: WT vs MTHFR-deficient mice at 11.35 kHz, *p* = 0.03). Supporting this finding, session-by-session analysis revealed that MTHFR-deficient mice exhibited a larger performance difference between easy and hard trials (Fig. 5e, t-test *p* = 0.02). This selective difference suggests that WT mice retained more precise frequency discrimination abilities under demanding perceptual conditions, whereas MTHFR-deficient mice may have been more affected by the subtle frequency difference in the challenging trials. This behavioral profile mirrors the imaging results: broadened cortical FRAs in MTHFR-deficient mice and the corresponding SVM-based reduction in neural separability selectively affected discrimination of nearby frequencies, while leaving coarse discrimination intact.

Our findings demonstrate that impaired one-carbon metabolism affects auditory function across development, from early-life acoustic communication to adult sound encoding and perception. Across various levels of analysis, MTHFR deficiency was associated with reduced auditory sensitivity and a selective loss of fine spectral resolution, while largely preserving coarse discrimination.

## Discussion

This study demonstrates that partial loss of MTHFR function disrupts auditory processing across development, linking early-life changes in vocal behavior to adult reductions in peripheral sensitivity and a selective impairment in fine spectral perception. Together, the data support a model in which one-carbon–related metabolic stress weakens cochlear output, engages compensatory central adjustments that preserve audibility, and ultimately limits perceptual fidelity under high-demand listening conditions.

In neonatal mice, ultrasonic vocalizations (USVs) provide a behavioral window into early auditory system maturation. MTHFR-deficient pups exhibited consistent shifts in USV structure—including lower start frequencies, altered call timing, and a redistributed call-type repertoire—observed in both males and females, with several features differing by sex. Notably, although the total number of calls remained constant across sessions, we observed genotype-specific shifts in spectral and temporal features. At P6, both WT and MTHFR-deficient pups produced a comparable number of calls during the first session, with no significant increase in the second, likely reflecting an early developmental stage in which strong dependence on maternal contact (e.g., for thermal regulation) drives elevated vocal output upon initial separation, and lack of potentiation, which develops towards the second postnatal week [14]. Despite this lack of vocal potentiation, the structure of the calls changed across sessions, and these changes diverged between genotypes. This divergence suggests that MTHFR deficiency could alter experience-dependent tuning driven by maternal reunion, biasing how vocal features are fine-tuned over time.

A mechanistic link between one-carbon metabolism and cochlear vulnerability has been previously suggested. MTHFR loss elevates homocysteine and reduces methylation capacity, both of which can cause cochlear vulnerability [4]. Prior work also links folate-deficient diets to threshold elevations [12, 22]. Consistent with this framework, our ABR data show that even heterozygous MTHFR loss elevates thresholds and reduces wave I amplitudes, indicating diminished auditory-nerve output. These findings are compatible with inner hair cell synaptopathy and/or afferent fiber dysfunction, though contributions from outer hair cell impairment remain possible. Future studies incorporating DPOAEs and synapse- and oxidative-stress markers (e.g., CtBP2, 4-HNE) will be important for localizing the primary lesion and defining how one-carbon disruption impacts cochlear microanatomy.

Reduced peripheral drive does not necessarily translate into uniformly degraded perception, because central circuits can partially compensate. Indeed, in the same adult animals in which ABRs revealed reduced cochlear output, coarse tone discrimination remained intact, indicating that basic perceptual separation of widely spaced frequencies can be preserved despite diminished afferent input. Notably, the magnitude of the ABR threshold elevation was substantially larger than the corresponding change in our perceptual performance metric, a pattern consistent with elevated central gain. Performance did decline selectively as frequencies approached the target tone, revealing a specific reduction in fine spectral acuity. This behavioral dissociation—coarse intact, fine impaired—suggests a partial compensatory mechanism that preserves gross discriminability while leaving fine-grained spectral resolution vulnerable.

Two-photon imaging in a separate cohort provided support for this hypothesis. Cortical imaging revealed robust tone-evoked responses in the auditory cortex, consistent with compensatory maintenance of cortical responsiveness. Yet this preserved response magnitude masked a qualitative change in cortical encoding: frequency–response areas were significantly broadened in MTHFR-deficient mice, indicating reduced frequency selectivity. In turn, decoding analyses showed reduced neural separability for closely spaced tones, while separability for widely spaced tones remained high. Although imaging and behavior were not performed in the same animals, the convergence between broadened cortical tuning, impaired decoding for nearby frequencies, and the behavioral vulnerability near the target tone supports a coherent account in which compensation stabilizes audibility while constraining fine spectral resolution.

Enhanced central gain is a plausible contributor to this tradeoff. In prior work, we observed auditory cortical hyperactivity in MTHFR-deficient mice [32], consistent with homeostatic upregulation in response to weakened peripheral drive. Such gain can stabilize the overall response magnitude and support detection or coarse discrimination; yet, it can also broaden tuning and increase overlap between population responses to nearby frequencies, thereby reducing the neural separability required for fine discrimination. In this view, MTHFR deficiency biases the system toward preserved audibility at the expense of spectral resolution when discrimination demands are high. This pattern is consistent with broader central-gain models in which sensorineural loss triggers compensatory amplification that can preserve audibility or coarse discrimination under simpler conditions, yet compromises fidelity (e.g., change in tuning/population separability) and therefore becomes most evident when perceptual demands are high [9, 10, 26, 29, 37].

An intriguing question is whether the severity of the early-life vocal anomalies can predict later perceptual deficits. While our neonatal and adult datasets were collected from separate cohorts, they likely reflect sequential stages of a shared pathological progression. In interpreting the neonatal phenotype, it is also important to note that WT pups were born to WT dams and Mthfr +/– pups were born to Mthfr +/– dams, so maternal environment/care could also contribute to group differences. Longitudinal studies tracking individuals from pup USVs to adult ABRs, cortical encoding, and perception —ideally incorporating cross-fostering and within-litter designs— will be critical for establishing whether neonatal “vocal fingerprints” can serve as early indicators of later-life auditory deficits, and for identifying the developmental window in which intervention might be most effective.

We began our study with both male and female neonates to capture the range of developmental phenotypes, and found genotype effects in both sexes with partially divergent expression. Given reports of sex-dependent metabolic and oxidative-stress responses in MTHFR-deficient mice [6, 8, 17, 21], we focused the adult arm of this study on males to reduce cohort heterogeneity. It remains an open question whether females show distinct adult physiological and perceptual outcomes, potentially reflecting different compensatory strategies or vulnerabilities. Addressing this explicitly will be important, both mechanistically and for translational relevance.

Finally, these findings may have broader relevance to neurodevelopmental conditions in which sensory phenotypes are prominent. In humans, MTHFR C677T polymorphism and folate-related metabolic disruption have been associated with increased risk for ASD and other neurodevelopmental outcomes [19, 25, 33]. Given the centrality of auditory cues for communication, even subtle reductions in auditory fidelity—especially those that evade standard threshold-based screening—could plausibly contribute to sensory challenges and, indirectly, to altered social interaction. Consistent with this possibility, altered social phenotypes have been reported in MTHFR-deficient mice [6, 30, 31]. Future work should directly test whether improving auditory fidelity mitigates aspects of these behavioral outcomes.

The clinical implications are also substantial. Our findings raise the possibility that mild metabolic disruption, often considered subclinical, could still degrade auditory fidelity in ways that elude standard audiometry but impair real-world listening. As recognition grows that suprathreshold deficits contribute to difficulties such as speech-in-noise perception, identifying metabolic contributors—and their modifiability through diet or supplementation—may offer a tractable route for early detection and intervention.

## Methods

### Experimental model and subject details

All procedures were approved by the Ben-Gurion University animal care and use committee. Data was collected from 21 pups (postnatal day 6) and 16 adult mice (8–16 weeks postnatal).

MTHFR-deficient mice were created by mating Mthfr ± (HT) [7] females with Balb/cAnNCrlBR background with GAD65-tdTomato males with C57/Bl6 BAC background [3]. Mice participating in the behavioral task were maintained on a reverse 12-h light/12-h dark cycle and provided ad libitum access to food and water.

### Ultrasonic vocalization (USV)

Acoustic recording: Ultrasonic signals were recorded using Avisoft Bioacoustics (Berlin, Germany) system including: UltraSoundGate 116 Hm with the Ultrasound Microphone CM16/CMPA, using the Avisoft Recorder 4.2.17 – Bioacoustics recording software. Recordings were set at a sampling frequency of 250 kHz in a trigger mode using a threshold of 0.5% of the signal's energy in the range of 10–250 kHz.

At postnatal day 6, 2–3 pups per litter were recorded. A total of 21 pups were included in the vocalization analysis: 8 male and 4 female Mthfr +/+ (WT) pups, born to Mthfr +/+ dams, and 3 male and 6 female Mthfr ± (MTHFR-deficient) pups, born to Mthfr ± dams.

Each pup was separated from the litter and placed in a transparent plastic cup (11 cm high and 10 cm in diameter) located on a warm pad. The microphone was placed 10 cm above the pup. After a 5-min isolation session (session 1), the pup was placed back in the home cage with the litter for 20 min and separated for a second recording session of 5 min (session 2), to further explore whether the experience of reunion with the dam impacts other properties of the pups' calls. The recording area was cleaned with Ethanol (70%) between pups.

USV Analysis: In order to analyze USV calls, we developed a designated algorithm based on spectrogram analysis. Briefly, the USV calls are divided into short segments of 0.5 s each. For each segment, the Fourier transform is calculated. Then, the energy of the signal for each frequency band is calculated and a pre-determined threshold is applied. The algorithm was implemented in Matlab and used to segment the USV calls, i.e., determine the beginning and end of each voice segment. Segmentation results were used to determine the time the USV was emitted, and the USV type was classified by a researcher blind to the group identity, based on the classification of 10 vocalization syllables as suggested before [7]. The following variables were used to compare USV syllables between groups: Start Frequency, defined as mean frequency at start of the syllable, End Frequency, defined as mean frequency at the end of the syllable, Duration defined by the time difference between start and end of the call and inter syllable interval (ISI) the time difference between the end of a call and the start of the following call, inter syllable intervals larger than 0.5 s were defined as inter burst intervals and not analyzed. Calls that were interrupted by noise or other recording problems were included only for the analysis of call quantity and call type but were omitted from the analysis of calls properties (frequencies, duration, and ISI). Total 12,550 calls were analyzed. 6184 male pups calls: 4471 calls emitted by WT and 1813 were emitted by Mthfr ± pups. 6366 female calls: 1963 calls emitted by WT and 4403 were emitted by Mthfr ± female pups.

### Auditory brainstem responses

Mice were anesthetized with ketamine (120 mg/kg) and xylazine (12 mg/kg), and placed on a homeothermic heating blanket during testing. ABR stimuli were 5-ms tone pips at 8,16, or 32 kHz with a 0.5-ms rise-fall time delivered at 30 Hz. Intensity was incremented in 5 dB steps, from 20–95 dB SPL. ABR threshold was defined as the lowest stimulus level at which a repeatable waveform could be identified. If we couldn’t find a repeatable waveform at the intensities tested, we defined the threshold as 100 dB for quantifications.

### Survival surgeries for awake, head-fixed imaging and behavior experiments

Mice were anesthetized with isoflurane in oxygen (5% induction, 1.5% maintenance). The dorsal surface of the mice's heads was trimmed and sterilized. ThermoStar homeothermic blanket monitoring system was used to maintain body temperature at 36.6C◦ (RWD). Lidocaine hydrochloride was administered subcutaneously to numb the scalp. The dorsal surface of the scalp was reduced using surgical scissors, and the periosteum was removed. The skull surface was prepped with an etchant (C&B metabond) and vetbond (3 M) before affixing a custom stainless-steel headplate to the dorsal surface with dental cement (C&B metabond). At the conclusion of the headplate attachment and any additional procedures listed below, Buprenex (0.05 mg/kg) and meloxicam (0.1 mg/kg) were administered, and the animal was transferred to a warmed recovery chamber.

### Virus mediated gene-delivery

For mice used in imaging experiments, two burr holes were made in the skull over the auditory cortex (1.75—2.25 mm rostral to the lambdoid suture and 4.0–4.5 mm lateral from the midline [5, 15, 16, 26, 28, 32]). A precision injection system (Nanoject III) was used to inject 75 nL of AAV5.Syn.GCaMP6s.WPRE.SV40 in each burr hole 200–250 mm below the pial surface. Before implanting the glass window, we waited ~ 3 weeks for virus incubation.

### Two-photon calcium imaging

Three round glass coverslips (one 4 mm, two 3 mm, #1 thickness) were etched with piranha solution and bonded into a vertical stack using transparent, UV-cured adhesive. Headplate attachment, anesthesia and analgesia follow the procedure described above. A 3 mm craniotomy was made over the right ACtx using a scalpel and the coverslip stack was cemented into the craniotomy. An initial widefield epifluorescence imaging session was performed to visualize the tonotopic gradients of the auditory cortex and identify the position of A1 as described previously [26, 28]. Two-photon excitation was provided by a Ti:Sapphire-pulsed laser tuned to 920 nm. Imaging was performed with a 16 X/0.8NA water-immersion objective (Nikon) from a 512X512 pixel field of view at 30 Hz with a Galvo-Resonant 8 kHz scanning microscope (Thorlabs). Scanning software was synchronized to the stimulus generation hardware using digital pulse trains. The microscope was rotated 50–60 degrees off the vertical axis to obtain images from the lateral aspect of the mouse cortex while the animal was maintained in an upright head position. Imaging was performed in a light-tight, sound-attenuating chamber mounted on a floating table. Animals were monitored throughout the experiment to confirm that all imaging was performed in the awake condition. Imaging was performed in layers L2/3, 200–230 mm below the pial surface. Fluorescence images were captured at 2 × digital zoom, providing an imaging field of (0.42 X 0.42 mm). Raw calcium movies were processed using Suite2P [23], a publicly available two-photon calcium imaging analysis pipeline. ∆F/F was computed as follows: (F(t)- F0)/F0, where F(t) was the raw calcium signal and F0 was the mean baseline fluorescence before stimulus presentation across trials.

### Auditory stimulus for imaging experiments

Auditory stimuli were generated with a 24-bit digital-to-analog converter (National Instruments model PXI-4461) using scripts programmed in MATLAB (MathWorks) and LabVIEW (National Instruments). Speakers were calibrated for their distance from the mouse's contralateral ear (left ear). For imaging experiments, we played 50 ms pure tones at different sound intensities (20–80 dB SPL with 10 dB SPL step) and frequencies (4 to 45 kHz with a half-octave step) combinations. All the trials had a 3-s duration. Each frequency-intensity combination was repeated 15 times.

### Two-photon calcium imaging analysis

#### Frequency response areas

The tone-evoked activity was calculated as the average activity in the 120 ms after the tone minus the average activity 120 ms before the tone. The tone-driven portion of the FRA of FRAs with a d-prime higher than 1.5 was used for the rest of the analysis and to determine the best frequency (BF), the frequency associated with the highest activity summed across all sound levels, and the FRA bandwidth measured 10 dB above the minimum response threshold. To characterize tuning quality (d-prime), 30 frequency-level combinations were sampled at random from the tone-driven and tone-unrelated portions of the raw FRA, and the mean spike counts of the two subsampled groups were calculated. This process was repeated 1000 times, yielding estimates of the distributions of the mean spike counts for both driven and undriven responses. The d-prime of the unit was defined as the difference between the means of these two distributions divided by their arithmetic average SD, reflecting the difference between the spike counts in the driven and undriven FRA regions relative to inherent variability. If the tone-driven portion of the FRA contained fewer than 50 tone-level combinations, additional random samples from the tone-unrelated portion were added to reach 50 samples (resulting in a conservative estimate of the unit responsiveness). Additional details on windowing and FRA boundary determination are described in Guo et al. [13]. For MTHFR-deficient mice, 34.3% of cells had FRAs with d-prime over 1.5, and for WT mice, it was 31.8%.

#### Frequency discrimination

We employed a linear kernel Support Vector Machine (SVM) classifier to determine how ensemble activity decoded the different frequencies at 70 dB SPL (well above threshold). This classifier model was trained using a data matrix of cell activity. The data matrix was comprised of the average sound-evoked activity within the FRA window after the stimulus onset, normalized by the pre-stimulus activity. Ensemble analysis included all cells per session (~ 140 cells per mouse on average). We employed k-fold cross-validation method (k = 5) to train the classifier and compute the misclassification rate on untrained trials (we got the same results with leave-one-out cross-validation). We replicated this procedure independently for each mouse and imaging session 100 times, ultimately calculating the mean decoding accuracy across sessions. The SVM training and cross-validation procedure were executed in MATLAB, utilizing the 'cvpartition', 'fitcsvm,' 'crossval,' and 'kfoldLoss' functions.

### Frequency discrimination task

Mice were water-restricted prior to behavioral training, maintaining 80–85% of their original body weight. During training sessions, pure tones (65 dB SPL) at varying frequencies were presented. Mice were trained to lick a water spout in response to a target tone (12 kHz) to receive a water reward and to withhold licking in response to all non-target frequencies. False alarms triggered a 4–6 s timeout penalty.

Training was conducted in two stages: first, mice were trained to associate the onset of a 12 kHz tone with licking the spout; second, they were trained to withhold licking in response to a 4 kHz tone. Once the mice reliably discriminated between these two tones, additional non-target frequencies closer to the target (6, 8.48, 10.09, 11.04, and 11.35 kHz) were introduced to further challenge frequency discrimination. Mice typically reached expert status after 26–32 training sessions. Non-target tones were 1 to 0.08 octaves away from the target, making the discrimination more challenging. Behavioral responses were classified as hits or false alarms based on licks occurring between 0.15 and 1.2 s following tone onset. Mice were tested for an average of 9 sessions.

### Statistical analysis

All statistical analyses were performed in MATLAB R2023a (Mathworks), Jamovi, and R. Data shown in all analyses is the mean activity ± SE unless otherwise indicated. Post hoc pairwise comparisons were corrected for multiple comparisons using the Bonferroni correction.

## Supplementary Information

Supplementary Material 1.

Supplementary Material 2.

Supplementary Material 3.

## Data Availability

All data will be available upon request.
