# Supplementary material for: Altered folate metabolism disrupts auditory function from neonatal vocalizations to adult perceptual precision
Source: J Neurodev Disord. 2026 Apr 10;18:29. doi: 10.1186/s11689-026-09692-2 (PMC13191893; doi:10.1186/s11689-026-09692-2)
Supplement: Supplementary file 3 — Supplementary Material 3. [file 11689_2026_9692_MOESM3_ESM.docx]

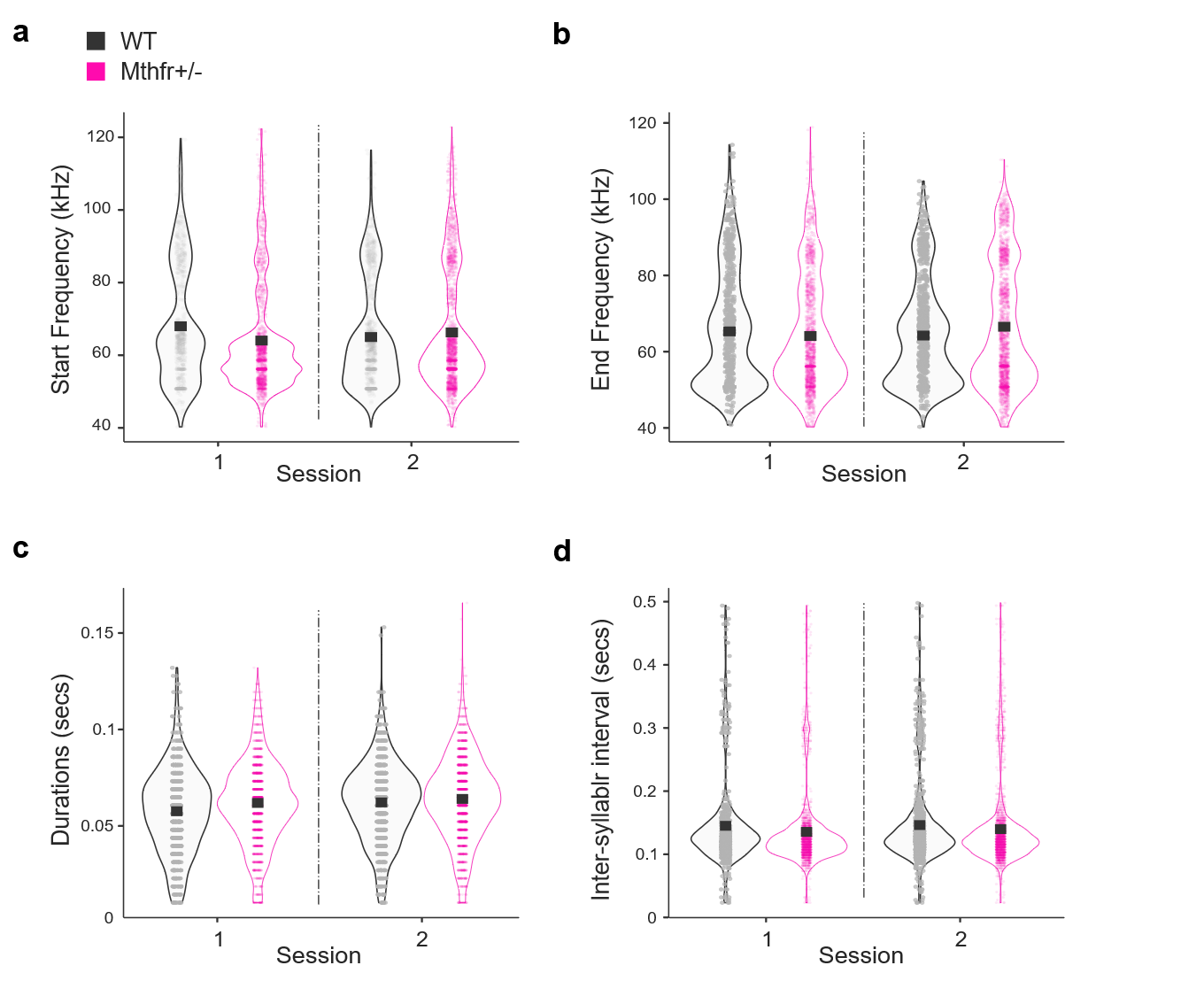


**Supplemental Figure 1**. **MTHFR-deficiency altered the spectral and temporal features of isolation calls in female mouse pups.**

1. Distribution of start frequencies for isolation calls emitted by 6 MTHFR-deficient (pink) and 4 wild-type (WT, black) pups. MTHFR-deficient pups initiated calls at significantly lower frequencies than WT pups (2-way ANOVA, genotype effect: F=8.6, p=0.00327).
2. Distribution of end frequencies. MTHFR-deficient pups exhibited slightly lower than WT in the first session but shifted upward in the second (2-way ANOVA genotype x session interaction F=15.670, p=0.00008).
3. Call duration was consistently longer in MTHFR-deficient pups across sessions (2-way ANOVA genotype F=24.470, p<0.00001).
4. Inter-syllable intervals (ISI) were significantly shorter in MTHFR-deficient pups (2-way ANOVA F=17.223, p=0.00003).

For panels (a–d), mean values are indicated by black squares.

**
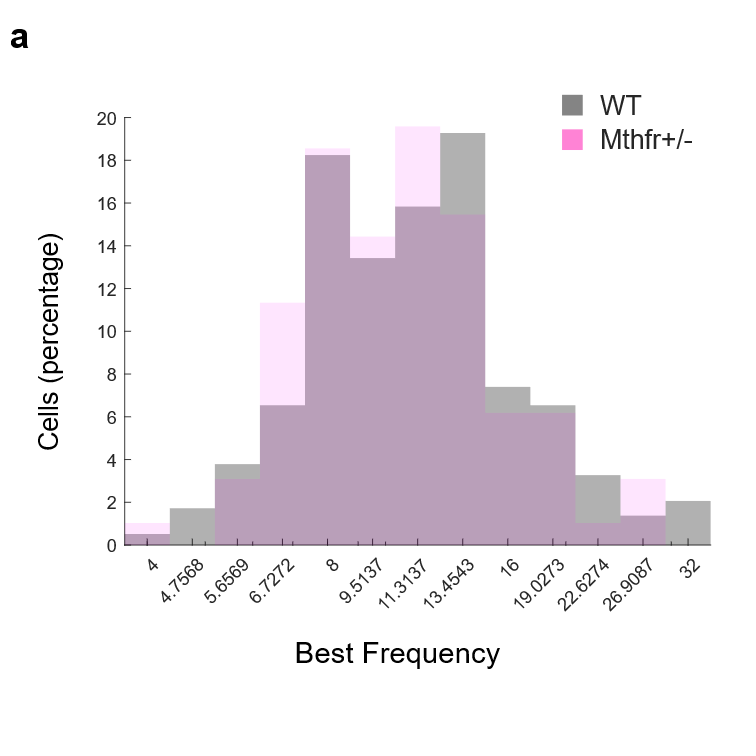
**

**Supplemental Figure 2**. **Best Frequency of imaged cells.**

Best frequency did not differ significantly between WT and MTHFR-deficient neurons with well-defined FRAs (d′ > 1.5; n = 170 WT cells, n = 154 MTHFR-deficient cells; t-test, p = 0.021).
